# Supplementary material for: Prevalence of malnutrition and associated factors among adult patients on antiretroviral therapy follow-up care in Jimma Medical Center, Southwest Ethiopia
Source: PLoS One. 2020 Mar 12;15(3):e0229883. doi: 10.1371/journal.pone.0229883 (PMC7067416; doi:10.1371/journal.pone.0229883)
Supplement: Attachment — Submitted filename: Reponse to Reviewers.docx [file pone.0229883.s003.docx]

January 21, 2020

**To:** The Editor, PLOS ONE

**Subject:** Response to the Editor’s and reviewer’s comments

We appreciate the comments provided by the editor and reviewers on the original research article submitted to PLOS ONE. In this letter we would like to provide our responses both for the comments provided by the editor and the reviewers separately.

Response to the Editor:

A per the comments provided by the Editor, we have modified the formats of the manuscript (the Title page and tables/figures) to be compatible with the PLOS ONE guidelines.

Regarding to the ethics statement, all patient’s data were initially anonymized in that we have accessed all patient records with only their unique ART number. Though, as this unique ART number might let identification of patients; patient data were collected by using unique codes for each records starting from 1001. As data were collected from secondary sources (patient records), we were unable to take informed consent from each patients. Instead, we have obtained a permission from authorities of the Medical Center based on the approval letter received from the Ethical Review Committee. The requirements of informed consent was waived by the IRB or ethics committee.

With regards to data sharing, we have included the de-identified or anonymized patient data into the supplementary information’s. The format of data included in the supplementary information’s was excel or spreadsheets.

Finally, the corresponding author have created ORCID iD and it was validated by Editorial manager.

Response to the reviewers*:*

Response to reviewer 1:

The main aim of this article was to estimate the prevalence of undernutrition and identify the predictors of undernutrition in adult HIV/AIDS patients on follow-up care. We have also estimated the prevalence of overweight or obesity among HIV/AIDS patients. This is the reason why we have defined the title as the prevalence of malnutrition instead of undernutrition and we repeatedly utilized the term malnutrition in the manuscript.

We have addressed the detail description of the study methods (study design, study population and participant’s selection processes) in the methods and materials section of the current or revised manuscript. In addition we have also clearly presented the study participation in the results section of the manuscript with support of the study flow diagram (Fig 1). Out of the total study participants (n=1062), normal nutrition, under nutrition and overweight or obesity accounted of 614, 357 and 91; respectively. The missing data (9%) in the previous version of the manuscript was observed as we didn’t clearly indicate the denominator in estimating overweight or obesity (lack of clarity in data presentation). Overall, in this version of the manuscript; we tried to adequately address the comments provided in the methods and results section by the reviewer.

As we have used secondary data for this specific article and only limited variables were included, we didn’t assess the food insecurity and wealth index of patients. Hence, the variables addressed were limited to the one presented in this article/manuscript.

We have addressed the comments provided in the results section. We have merged table 1, 2 and 3. Hence, the total number of tables in the manuscript was reduced to 3. We have also incorporated the comments provided in the ‘Prevalence of malnutrition’ sub-heading of the Results section of the manuscript. Additionally, we have also addressed the minor comments provided by the reviewer.

Response to reviewer 2:

Most of the comments provided by the reviewer are related to the clarity of the method section and language. In the revised version of the manuscript, we have clearly presented the study methods (including the study design, study population, sampling, inclusion/exclusion criteria and analysis). We have also revised the language.

Kindly regards,

Dawit Wolde Daka

Correspondence author
